# Gene-expression analysis of gleason grade 3 tumor glands embedded in low- and high-risk prostate cancer

**DOI:** 10.18632/oncotarget.9344

**Published:** 2016-05-13

**Authors:** A. Marije Hoogland, René Böttcher, Esther Verhoef, Guido Jenster, Geert J.L.H. van Leenders

**Affiliations:** ^1^ Departments of Pathology, Erasmus Medical Center, Rotterdam, The Netherlands; ^2^ Departments of Urology, Erasmus Medical Center, Rotterdam, The Netherlands; ^3^ Department of Bioinformatics, University of Applied Sciences Wildau, Wildau, Germany

**Keywords:** prostate cancer, RNA sequencing, laser capture microdissection, immunohistochemistry

## Abstract

The Gleason score (GS) of prostate cancer on diagnostic biopsies is an important parameter for therapeutic decision-making. Biopsy GS under-estimates the actual GS at radical prostatectomy in a significant number of patients due to sampling artifact. The aim of this study was to identify markers that are differentially expressed in Gleason grade 3 (GG3) tumor glands embedded in GS 4 + 3 = 7 and GS 3 + 3 = 6 prostate cancer using laser capture microdissection and RNA sequencing.

GG3 tumor glands embedded in nine GS 3 + 3 = 6 and nine GS 4 + 3 = 7 prostate cancers were isolated by laser capture microdissection of frozen radical prostatectomy specimens. After RNA amplification and RNA sequencing, differentially expressed genes in both GG3 components were identified by a 2log fold change > 1.0 and *p*-value < 0.05. We applied immunohistochemistry on a tissue micro-array representing 481 radical prostatectomy samples for further validation on protein level.

A total of 501 genes were up-regulated and 421 down-regulated in GG3 glands embedded in GS 4 + 3 = 7 as compared to GS 3 + 3 = 6 prostate cancer. We selected *HELLS, ZIC2* and *ZIC5* genes for further validation. ZIC5 mRNA was up-regulated 17 fold (*p* = 8.4E–07), ZIC2 8 fold (*p* = 1.3E–05) and HELLS 2 fold (*p* = 0.006) in GG3 glands derived from GS 4 + 3 = 7. HELLS expression of ≥ 1% occurred in 10% GS < 7, 17% GS 7 and 43% GS >7 prostate cancer (*p* < 0.001). Using a cut-off of ≥ 1%, protein expression of ZIC5 was present in 28% GS < 7, 43% GS 7 and 57% GS > 7 cancer (*p* < 0.001). ZIC2 was neither associated with GS nor outcome in our validation set. HELLS was independently predictive for biochemical-recurrence after radical prostatectomy (HR 2.3; CI 1.5–3.6; *p* < 0.01).

In conclusion, HELLS and ZIC5 might be promising candidate markers for selection of biopsy GS 6 prostate cancer being at risk for up-grading at prostatectomy.

## INTRODUCTION

With approximately 260,000 deaths per year worldwide, prostate cancer is a leading cause of cancer morbidity and mortality [1–3]. Prostate cancer demonstrates a highly variable disease course with many patients having asymptomatic disease. Pathologic grading of prostate cancer according to the Gleason grading system [4] is an important parameter for therapeutic decision-making [5]. The Gleason grading system has been used since the 1960s and is entirely based on tumor growth patterns. Increasing Gleason score (GS) is strongly associated with adverse histopathological and clinical endpoints, including pathologic stage, metastatic disease and survival [5]. Nevertheless, the prediction of tumor behavior and determination of optimal treatment modalities for GS 6 and 7 prostate cancer patients are uncertain [6–19].

While GS 6 prostate cancer demonstrates an indolent disease course in many patients, 55–90% of patients still undergo radical prostatectomy [20]. A widely used alternative to surgery is active surveillance. Up to 33% of patients on active surveillance, however, will eventually undergo therapeutic intervention after a median follow-up of 1–4 years [10, 11, 21–23]. Therefore, more accurate stratification of GS 6 prostate cancer patients is needed. A caveat of current Gleason grading on diagnostic biopsies is sampling artifact. Up to 38% of the patients with GS 6 prostate cancer on biopsy has GS ≥ 7 at subsequent radical prostatectomy [24, 25]. To optimize treatment decisions, it is essential to identify patients at risk for tumor under-grading at diagnosis.

GS 7 prostate cancer is composed of a mixture of regular tumor glands (Gleason grade 3) and aberrant glandular tumor structures (Gleason grade 4). Due to tumor heterogeneity, diagnostic biopsies might only sample Gleason grade 3 tumor areas leaving clinically relevant Gleason grade 4 patterns undiscovered. We hypothesize that Gleason grade 3 tumor glands embedded in GS 7 prostate cancer have different molecular expression profiles than Gleason grade 3 tumor glands embedded in GS 6 cancer.

The aim of this study was to identify markers that are differentially expressed in Gleason grade 3 tumor glands embedded in GS 4 + 3 = 7 and GS 3 + 3 = 6 prostate cancer using laser capture microdissection and RNA sequencing.

## RESULTS

### Gene-expression analysis

For each sample, RNA sequencing reads of 101 nt were generated in paired-end fashion, which yielded 27–130 million sequencing reads per sample (Table 1). Mapping rates between samples were comparable (58.6–73.2% of total reads mapped, of which 63.2–89.8% were mapped uniquely to the genome) and in total, between 2.4 and 19 million reads could be uniquely assigned to RefSeq genes. At a logCPM cut-off ≥ 1, 8133 out of the total 23.373 annotated genes were detected. The workflow of the RNA sequence data was performed both on the CLC workbench, as well as TopHat and RefSeq, leading to approximately 80% of overlap in genes.

**Table 1:** Mapping statistics

sample	Gleason grade	total Gleason score	Total raw reads^*^	mapped reads	% mapped
EMC_1	3	3 + 3 = 6	91792508	35616115	0.388006775
EMC_3	3	3 + 3 = 6	80480262	33218341	0.412751402
EMC_5	3	3 + 3 = 6	61361596	24674668	0.402119071
EMC_11	3	4 + 3 = 7	44494648	15483302	0.347981222
EMC_13	3	3 + 3 = 6	27359166	9515977	0.347816779
EMC_14	3	4 + 3 = 7	46561926	18944840	0.406874063
EMC_15	3	4 + 3 = 7	39994636	14221623	0.355588259
EMC_16	3	3 + 3 = 6	75881474	28856468	0.38028344
EMC_17	3	3 + 3 = 6	59607656	24015364	0.402890595
EMC_18	3	4 + 3 = 7	39222968	13941906	0.355452601
EMC_19	3	4 + 3 = 7	92965194	40163311	0.432025248
EMC_21	3	4 + 3 = 7	82403308	35362238	0.429136146
EMC_23	3	3 + 3 = 6	90405938	38248796	0.42307836
EMC_24	3	3 + 3 = 6	108139494	47354477	0.437901781
EMC_25	3	4 + 3 = 7	87480724	37809613	0.432205076
EMC_27	3	4 + 3 = 7	80044030	33613406	0.419936452
EMC_28	3	3 + 3 = 6	89640362	37496355	0.418297675
EMC_29	3	4 + 3 = 7	92211070	40098913	0.434860077
EMC_1	14127736	21488379	0.396666958	0.603333042	10410480
EMC_3	12283062	20935279	0.369767473	0.630232527	9584359
EMC_5	9840075	14834593	0.3987926	0.6012074	4247549
EMC_11	7232723	8250579	0.467130526	0.532869474	2399724
EMC_13	4050156	5465821	0.425616413	0.574383587	3659443
EMC_14	7724933	11219907	0.40775921	0.59224079	4274365
EMC_15	5193919	9027704	0.365212817	0.634787183	3869535
EMC_16	11777779	17078689	0.408150401	0.591849599	4928250
EMC_17	9533194	14482170	0.396962295	0.603037705	6221822
EMC_18	4964691	8977215	0.356098442	0.643901558	5967889
EMC_19	12237918	27925393	0.304703912	0.695296088	17445060
EMC_21	11498196	23864042	0.325154647	0.674845353	12957564
EMC_23	12212986	26035810	0.319303802	0.680696198	15348892
EMC_24	14733626	32620851	0.311134806	0.688865194	15492077
EMC_25	11978513	25831100	0.316811309	0.683188691	13804904
EMC_27	12591218	21022188	0.374589174	0.625410826	5594209
EMC_28	13153841	24342514	0.350803191	0.649196809	12254753
EMC_29	12709260	27389653	0.316947744	0.683052256	13348694

* both strands included.

UMR = uniquely mapping reads. MMR = multi-mapping reads.

With regard to the limited number of samples, we determined the genes that were up-regulated in Gleason grade 3 derived from GS 4 + 3 = 7 prostate cancer as compared to Gleason grade 3 glands from GS 3 + 3 = 6 tumors with a 2log fold change > 1.0 and *p* < 0.05. A total of 501 genes were over-expressed in Gleason grade 3 tumor glands being part of a GS 4 + 3 = 7 as compared to GS 3 + 3 = 6 prostate cancer and 421 genes were down-regulated (Supplementary Tables 1 and 2). We selected the genes helicase, lymphoid specific (HELLS), and zinc finger of the cerebellum (ZIC) family members 2 and 5 for further validation. Selection of candidates was based on a) up-regulation in Gleason grade 3 in a GS 4 + 3 = 7 prostate cancer, b) commercial availability of a reliable antibody, c) novelty in clinical prostate cancer, d) staining reliability and heterogeneity on radical prostatectomy slides, and e) nuclear protein location. ZIC5 mRNA was up-regulated 17 fold (*p* = 8.4E–07), ZIC2 8 fold (*p* = 1.3E–05) and HELLS 2 fold (*p* = 0.006) in Gleason grade 3 tumor glands derived from GS 4 + 3 = 7 as compared to GS 3 + 3 = 6 prostate cancer.

### Immunohistochemical expression

**Figure 1:**
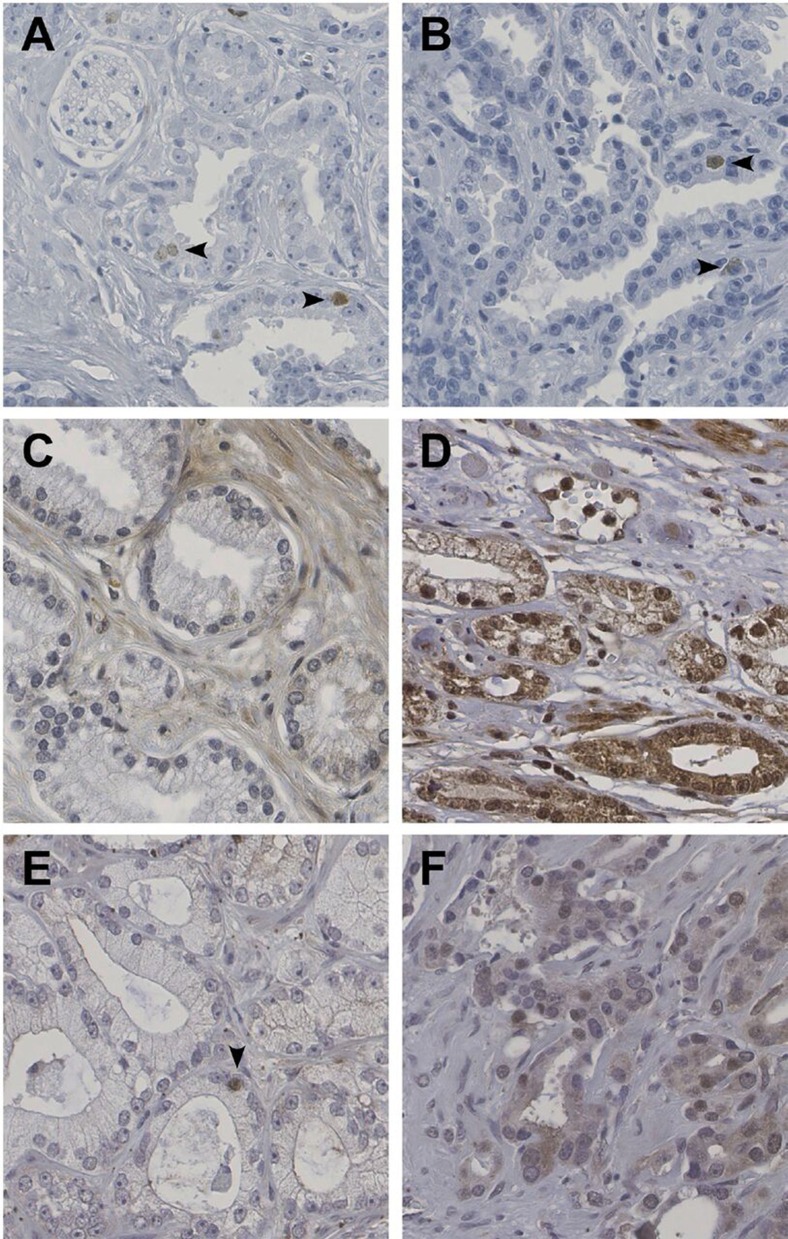
Immunohistochemical expression of HELLS (A, B), ZIC2 (C, D) and ZIC5 (E, F) in prostate cancer HELLS is positive in a small subpopulation of tumor cells (arrowheads). ZIC2 is negative (C) or moderately positive in a high percentage of cells with both cytoplasmatic and nuclear expression (D). ZIC5 shows sporadic (E) or moderate (F) nuclear expression in scattered cells. Original magnifications, 400×.

Staining of HELLS was observed in the nuclei of lymphocytes (positive control) and was not expressed in placental tissues (negative control). No immunohistochemical staining of HELLS was observed in the nuclei of luminal or basal cells in benign prostate glands. Nuclear staining of HELLS was observed in 162 out of 421 patients (39%), with the percentage of positive tumor nuclei varying between 0.2% and 9.3% (Figure 1A and 1B). A total of 64 out of 421 patients (15%) showed HELLS staining in ≥ 1% of tumor cells. HELLS expression of ≥ 1% was significantly associated with GS (*p* < 0.001; Table 1); 23 out of the 221 patients with GS < 7 (10%) expressed HELLS as compared to 29 out of the 172 with GS 7 (17%) and 12 out of 28 patients with GS > 7 (43%). HELLS was expressed in > 1% of tumor cells in 8/30 (27%) GS 4 + 3 = 7 cases as compared to 20/142 (14%) of GS 3 + 4 = 7 tumors (*p* = 0.09). In addition, HELLS was independently predictive for biochemical recurrence (HR 2.3; CI 1.5–3.6; *p* < 0.001) after radical prostatectomy adjusted for including age, PSA, GS, pT-stage and surgical margin status in multivariate analysis (Table 2). HELLS was neither associated with PSA (*p* = 0.71) nor pT-stage (*p* = 0.07).

**Table 2:** Correlation of HELLS, ZIC2 and ZIC5 with clinicopathologic parameters

HELLS				
	Negative	Positive	Total	*p*-value
PSA at diagnosis				0.71
≤ 10 ng/ml	312 (74.3%)	55 (13.1%)	367 (87.4%)	
> 10 ng/ml	44 (10.5%)	9 (2.1%)	53 (12.6%)	
Total	356 (84.8%)	64 (15.2%)	420	
Gleason score				< 0.001
< 7	198 (47.0%)	23 (5.5%)	221 (52.5%)	
7	143 (34.0%)	29 (6.8%)	172 (40.8%)	
> 7	16 (3.8%)	12 (2.9%)	28 (6.7%)	
Total	357 (84.8%)	64 (15.2%)	421	
pT-stage				0.07
pT2	257 (61.1%)	37 (8.8%)	294 (69.9%)	
pT3a/b	81 (19.2%)	21 (5.0%)	102 (24.2%)	
pT4	19 (4.5%)	6 (1.4%)	25 (5.9%)	
Total	357 (84.8%)	64 (15.2%)	421	

ZIC2				
	Negative	Positive	Total	*p*-value
PSA at diagnosis				0.66
≤ 10 ng/ml	176 (52.9%)	108 (32.4%)	284 (85.3%)	
> 10 ng/ml	32 (9.6%)	17 (5.1%)	49 (14.7%)	
Total	208 (62.5%)	125 (37.5%)	333	
Gleason score				0.18
< 7	98 (29.5%)	61 (18.3%)	159 (47.8%)	
7	88 (26.4%)	58 (17.4%)	146 (43.8%)	
> 7	22 (6.6%)	6 (1.8%)	28 (8.4%)	
Total	208 (62.5%)	125 (37.5%)	333	
pT-stage				0.77
pT2	136 (40.9%)	83 (24.9%)	219 (65.8%)	
pT3a/b	58 (17.4%)	36 (10.8%)	94 (28.2%)	
pT4	14 (4.2%)	6 (1.8%)	20 (6.0%)	
Total	208 (62.5%)	125 (37.5%)	333	

ZIC5				
	Negative	Positive	Total	*p*-value
PSA at diagnosis				0.13
≤ 10 ng/ml	237 (57.4%)	122 (29.5%)	359 (86.9%)	
> 10 ng/ml	30 (7.3%)	24 (5.8%)	54 (13.1%)	
Total	267 (64.7%)	146 (35.3%)	413	
Gleason score				< 0.001
< 7	161 (38.9%)	61 (14.7%)	222 (53.6%)	
7	94 (22.7%)	70 (16.9%)	164 (39.6%)	
> 7	12 (2.9%)	16 (3.9%)	28 (6.8%)	
Total	267 (64.5%)	147 (35.5%)	414	
pT-stage				0.25
pT2	193 (46.6%)	95 (23.0%)	288 (69.6%)	
pT3a/b	58 (14.0%)	42 (10.1%)	100 (24.1%)	
pT4	16 (3.9%)	10 (2.4%)	26 (6.3%)	
Total	267 (64.5%)	147 (35.5)	414	

For HELLS and ZIC5 a cut-off of 1% was applied, for ZIC2 a cut-off of 50%.

The ZIC2 staining pattern demonstrated both cytoplasmatic and nuclear staining in most cases. As positive control, tubular epithelium in human kidney was used, and placenta tissue was used as negative control. In normal prostate glands, ZIC2 staining was observed in basal cells, as well as in luminal cells. In our cohort, 315 cases could be scored for ZIC2 staining; cytoplasmic staining was seen with weak intensity in 197 (63%) and moderate intensity in 19 (6%) patients. Nuclear staining intensity varied from negative in 130 (41%), weak staining in 109 (35%), moderate staining in 69 (22%) and strong staining in 7 (2%) patients (Figure 1C and 1D). ZIC2 protein expression (Table 2) was not associated with PSA (*p* = 0.66), GS (*p* = 0.18) or pT-stage (*p* = 0.77). ZIC2 did not have predictive value for biochemical recurrence after radical prostatectomy.

For ZIC5, basal cells in normal prostate glands were used as positive control tissue, whereas placental tissue served as negative control. Overall ZIC5 staining intensity was low. Of the 414 evaluable cases, 147 (36%) demonstrated nuclear staining of ZIC5. In 119 (29%) patients staining was weak, in 26 (6%) patients staining was moderate and in 2 (0.4%) strong. One-hundred patients (24%) showed nuclear ZIC5 staining in < 1% of the tumor cells, 32 patients (8%) expressed ZIC5 in 1–5% of the tumor cells and 15 patients (4%) expressed ZIC5 in > 5% of the tumor cells (Figure 1E and 1F). Using a cut-off of ≥ 1%, protein expression of ZIC5 was associated with GS (*p* < 0.001; Table 1); 61 out of 222 patients with GS < 7 (28%) showed ZIC5 protein expression as compared to 58 out of 146 with GS 7 (43%), and 16 out of 28 with GS > 7 (57%). ZIC5 was expressed in > 1% of tumor cells in 15/29 (52%) of evaluable GS 4 + 3 = 7 patients and 55/135 (41%) of GS 3 + 4 = 7 patients (*p* = 0.28). There was no significant association of ZIC5 expression and PSA (*p* = 0.13) or pT-stage (*p* = 0.25). ZIC5 expression did not have predictive value for biochemical recurrence after operation (Table 3).

**Table 3:** Predictive value of HELLS, ZIC2 and ZIC5 for biochemical recurrence after radical prostatectomy

Parameter	Univariate analysis		Multivariate analysis	
	HR (95% CI)	*p*-value	HR (95% CI)	*p*-value
Age	1.0 (1.0–1.09)	0.04	1.0 (1.0–1.1)	0.21
PSA	3.5 (2.3–5.3)	< 0.01	1.8 (1.1–2.8)	0.03
Gleason score	2.5 (1.9–3.3)	< 0.01	1.8 (1.3–2.5)	< 0.01
pT-stage	1.7 (1.5–2.0)	< 0.01	1.3 (1.0–1.5)	0.03
Surgical margin	3.3 (2.3–4.7)	< 0.01	2.3 (1.6–3.4)	< 0.01
				
HELLS	2.6 (1.7–4.0)	< 0.01	2.3 (1.5–3.6)	< 0.01
ZIC2	1.1 (0.7–1.7)	0.69	1.4 (0.9–2.2)	0.11
ZIC5	1.3 (0.9–1.9)	0.15	0.9 (0.6–1.3)	0.61

HR = Hazard ratio; CI = Confidence Interval; PSA = Prostate Specific Antigen.

Since HELLS is known to be involved in regulation of DNA methylation as well as histone modification, we analysed the relation of HELLS with Polycomb histone methyltransferase protein EZH2 [26]. Previously, we had shown that EZH2 expression in ≥ 1% of tumor cells was associated with high GS and biochemical recurrence [27, 28]. As we had determined EZH2 protein expression on the similar cohort, we analysed the expression of HELLS and EZH2 in similar tumor areas [27]. Using a cut-off of 1% for both HELLS and EZH2, we found a strong association between both proteins in the same patients (*p* = 0.004) and individual tissue-microarray punch cores (*p* = 0.03).

## DISCUSSION

A major challenge in prostate cancer management is the decision on the optimal therapeutic approach in GS 6 and 7 patients. The ERSPC has shown that prostate cancer screening can result in 20% risk reduction in disease-specific mortality, however, at the cost of significant overtreatment [29]. On the other hand, 40% of GS 6 prostate cancer patients under surveillance are switched to deferred treatment after a median of 7.5 years [30]. These results clearly demonstrate that better risk stratification is needed for GS 6 and 7 prostate cancer patients.

Current therapeutic stratification of prostate cancer patients predominantly relies on serum PSA level, biopsy GS and number of positive biopsies. Systematic biopsies might however, not be representative for the entire tumor and can lead to discordance of GS at diagnostic biopsy and radical prostatectomy. Fine and Epstein demonstrated that 20% patients with GS 6 at biopsy were up-graded to GS 7 (18.7%) or 8–10 (1.3%) at radical prostatectomy [31]. Helpap and Egevad found concordance between GS 6 prostate cancer at biopsies and radical prostatectomies in only 28% [32]. The odds for up-grading are influenced by prostate volume, total number of biopsies taken, number of positive biopsies and biopsy tumor extent [33–35]. The objective of the current study was to identify differentially expressed genes in glandular Gleason grade 3 being part of GS 3 + 3 = 6 and GS 4 + 3 = 7 prostate cancer.

For this purpose, we isolated Gleason grade 3 tumor glands from a total of 18 patients using LCM and analyzed gene-expression values after RNA sequencing. Since the numbers of samples in both groups were small (total *N* = 18), we determined the genes with a 2log-fold chance > 1 and *p* < 0.05, resulting in 501 genes over-expressed and 421 genes down-regulated in Gleason grade 3 component of GS 3 + 3 = 6 as compared to GS 4 + 3 = 7 prostate cancer. The differentially expressed gene-list contained various genes previously reported to be differentially expressed in low- and intermediate/high-grade prostate cancer such as HOXC6, MAGEC2, HERPUD1 and SATB1 [36–39]. From the differentially expressed genes, we selected ZIC2, ZIC5 and HELLS for further validation on protein level in an independent cohort of 481 radical prostatectomy samples. Interestingly, expression of ZIC2, ZIC5 and HELLS protein was generally low indicating a discordance between mRNA and protein levels. Of these three genes, HELLS and ZIC5 protein expression was strongly associated with GS ≥ 7 prostate cancer, while we were not able to confirm this relation on protein level for ZIC2. In addition, HELLS was an independent predictive marker for biochemical recurrence after radical prostatectomy.

The *zinc finger of the cerebellum* (ZIC) family of genes encompasses five human homologues ZIC1–5 [40]. The family members are all transcription factors which have predominantly been implicated in neuro-ectodermal development [41]. ZIC family members are able to inhibit TCF4/β-Catenin and interact with GLI signaling [42, 43]. In addition, ZIC family members are involved in various human solid tumors. ZIC1, −2 and −5 are over-expressed in meningiomas, while ZIC4 is over-expressed in medulloblastomas [44]. High expression of ZIC2 is associated with survival in oral squamous carcinoma [45]. Methylation of ZIC4 in pTa bladder cancer is predictive for progression to muscle-invasive (≥ pT2) disease [46]. Promotor methylation of ZIC1 is associated with gastric cancer [47]. Interestingly, we found that ZIC1, ZIC2, ZIC4 and ZIC5 were all up-regulated in Gleason grade 3 embedded in GS 4 + 3 = 7 prostate cancer on RNA level. While high ZIC5 was associated with GS ≥ 7 on protein level, we were not able to demonstrate this for ZIC2.

The *helicase, lymphoid specific* (*HELLS*) gene also known as *lymphoid-specific helicase* (*LSH*) is known to activate E2F-transcription factors in G1/S-transition, and recruit DNA repair and chromatin-remodeling proteins [48]. HELLS has been implicated in the progression in non-small cell lung carcinoma and squamous cell carcinoma in the head and neck region [49–51]. Von Eyss et al. found that HELLS over-expression was associated with GS ≥ 4 + 3 = 7 tumors in 47 prostate cancer biopsies, which is in line with our study [52]. During development, HELLS is involved in silencing of HOX genes by recruitment of DNA methyltransferases and Polycomb-repressive complex members among which EZH2 [26]. Interestingly, EZH2 demonstrates similar expression patterns in prostate cancer as HELLS with expression in a minority of tumor cells. Using a cut-off of ≥ 1% in radical prostatectomy specimens, we found that EZH2 expression was associated with high-grade prostate cancer and biochemical recurrence [27, 28]. EZH2 expression in < 1% of tumor cells in prostate cancer biopsies was predictive for indolent disease at radical prostatectomy [19]. Although we did not perform immunofluorescent co-expression studies, we found statistical correlation of HELLS and EZH2 expression within the same patients and individual tissue microarray cores, suggesting a link of both DNA- and histone-methylation pathways within prostate cancer patients as has previously been shown in *in vitro* models [26].

In this study, we have analyzed gene-expression profiles in Gleason grade 3 in GS 3 + 3 = 6 and GS 4 + 3 = 7 prostate cancer using LCM, RNA amplification and sequencing. Hereby, we demonstrate the feasibility of this methodology for in-depth analysis of specific cellular tissue compartments. Our validation of a selected panel of genes by QPCR and the differential expression of various known genes such as HOXC6 and HERPUD1 supports the validity of this methodology. Using this approach, we were able to identify genes of interest that might have been undetected due to background signal when conventional whole tissue slide profiling is applied.

A caveat of the current study was the relatively low number of samples in both cohorts, which hampered robust biostatistical analysis. Therefore, we set less stringent discriminatory criteria for comparing both study groups and selected a panel of genes for further validation. We applied immunohistochemistry for validation of differential gene-expression profiles and did not include a validation by QPCR on independent laser microdissected patients samples. While our purpose was to validate genes such as HELLS and ZIC5 immunohistochemically on diagnostic GS 3 + 3 = 6 biopsies to select patients with actual GS ≥ 7 disease, biopsy validation was not included in the current study. Since retrospective biopsy studies are hampered by the fact that a significant number of biopsies will not have representative tissue available anymore, we are currently analyzing the predictive value of HELLS and ZIC5 in a prospective biopsy study.

In conclusion, we demonstrated that combining LCM, RNA amplification and sequencing resulted in reliable molecular profiling of specific tissue compartments. We found that in particular ZIC family members are over-expressed in Gleason grade 3 tumor glands associated with GS 4 + 3 = 7 disease. ZIC5 and HELLS were associated with GS ≥ 7 prostate cancer, while HELLS was independently predictive for post-operative biochemical recurrence.

## MATERIALS AND METHODS

### Clinical specimens

We selected 18 radical prostatectomy specimens of men who had been operated for prostate cancer at Erasmus Medical Center between 2005 and 2011. Directly after surgery, the prostate was transported on ice to the pathology department where a transverse tissue slide was snap frozen in liquid nitrogen for research purposes. The remaining prostate was injected with neutral-buffered formalin (4%) to allow for fast and equal fixation, and processed for routine pathologic diagnosis. For this study, we selected nine patients with GS 3 + 3 = 6 prostate cancer and nine patients with GS 4 + 3 = 7. Selection was based on availability of tissues and estimated tumor percentage in the frozen tissue. Reference hematoxylin/eosin (HE) staining was performed on the frozen tissue samples to verify the Gleason grade and estimate the tumor's percentage.

For validation purposes, we used tissue microarrays containing samples of 481 radical prostatectomy specimens in triplicate, as described previously [27, 53, 54]. Clinical follow-up was recorded after each control visit at our outpatient clinic and data were transmitted to the central study database. Post-operative biochemical recurrence was defined as an increase in serum Prostate Specific Antigen (PSA) after two different measurements, at least three months apart. The use of tissue samples for scientific purposes was approved by institutional board review (MEC-2011-295, MEC-2011-296). Samples were used according to the “Code for Proper Secondary Use of Human Tissue in The Netherlands” as developed by the Dutch Federation of Medical Scientific Societies (FMWV, version 2002, update 2011).

### Laser capture microdissection

For laser capture microdissection (LCM), we cut 10 μm thick tissue sections from the frozen tumor slices. After every two 10 μm thick slide, a 5 μm thick slide was made for HE reference evaluation. Each section was mounted on a glass slide (MembraneSlide, 1.0 mm PEN-membrane covered; Zeiss Micro-Imaging GmbH, Munich, Germany) and air-dried for 10 minutes. All glass slides had been treated with UV-light for 30 minutes. After drying, each glass slide was individually packed in tinfoil and stored at −80°C until further use. All materials and the cryostate had been cleaned with 70% ethanol prior to tissue handling.

For LCM, each slide was individually taken out of −80°C storage, unwrapped and immersed in RNA *later* Solution (Stabilization Solution; Ambion, UK). Slides were stained with Mayer's Hematoxylin for 30 seconds and dehydrated in a 50%–100% ethanol/MilliQ H_2_O gradient. Slides were air-dried and dissected at the PALM laser capture microdissection microscope (PALM Axio Observer A1, Zeiss Micro-Imaging, Munich, Germany) for a maximum of 60 minutes at room temperature. Individual tumor glands with Gleason grade 3 were carefully selected and dissected, to prevent contamination with surrounding normal stromal tissue or other tumor grade components. Dissected elements were automatically catapulted into the lid of a 500 μl eppendorf (AdhesiveCap opaque; Zeiss Micro-Imaging, Munich, Germany). 65 μl RLT+ buffer (from Qiagen AllPrep DNA/RNA Micro Kit; Qiagen, Venlo, The Netherlands) was added and samples were stored at −80°C until further use.

### RNA isolation

After dissection and storing, the 10 individually dissected samples from each patient were pooled resulting in 9 samples with Gleason grade 3 tumor glands derived from GS 3 + 3 = 6 prostate cancer, and 9 Gleason grade 3 samples derived from GS 4 + 3 = 7 prostate cancer. The total amount of RLT+ buffer after pooling was complemented to 650 μl. For RNA isolation the Qiagen AllPrep micro kit was used (Qiagen AllPrep DNA/RNA Micro Kit; Qiagen, Venlo, The Netherlands) according to the manufacturer's guidelines. RNA quality and quantity was measured using the Nanodrop Spectrophotometer (Model ND-1000, NanoDrop Technologies, Wilmington, USA) and Bioalalyzer Nanochip or Picochip (RNA 6000 Nano/Pico Kit, Agilent Technologies, Waldbronn, Germany). The average RNA amount harvested for the GS 3 + 3 = 6 group was 80.5 ng (2.9–240.0 ng) with RNA Integrity Number (RIN) values between 1.0 and 7.0. For the Gleason grade 3 samples derived from GS 4 + 3 = 7 prostate cancer, the average RNA amount was 35.4 ng (10.3–76.7 ng) with RIN values between 1.0 and 5.2.

### RNA sequencing

RNA amplification and sequencing was performed by AROS (AROS Applied Biotechnology A/S, Aarhus, Denmark). Amplification was performed using NuGEN's Ovation RNA-seq v2 System to generate double stranded cDNA. A control sample (10 ng of human reference RNA) was included. All samples produced similar amounts of double stranded cDNA (measured with the Qubit BR dsDNA kit). 1.2 μg of the dsDNA was used as input for the DNA TruSeq library prep to produce the library.

### Data workflow

All RNA sequence data were aligned to pre-indexed human reference genome (hg19, available via the bowtie2 homepage including annotation) using TopHat version 2.0.4 [55]. To increase accuracy, reads were aligned against the indexed transcriptome prior to alignment to the genome via setting “—transcriptome-index”. Other specified TopHat2 settings were “—b2-very-sensitive —report-secondary-alignments —read-realign-edit-dist 1—mate-inner-dist 200 —mate-std-dev 50”. RNA expression levels were quantified via HTSeq-count (http://www-huber.embl.de/users/anders/HTSeq, version 0.5.4p1) using the UCSC hg19 annotation provided by the TopHat2 developers. Subsequently, we used edgeR (version 3.0.4) to investigate differentially expressed genes between our different Gleason grade groups [56]. For internal validation purposes, we additionally performed RNA-sequence analysis using CLC Genomics Workbench version 5.1 (Qiagen, Venlo, The Netherlands). We followed the outlined RNA-sequence analysis pipeline and used hg19 as reference genome. All TopHat2 alignments were performed on the High Performance Cloud (https://www.surfsara.nl/systems/hpc-cloud). Downstream analyses as well as the CLC Genomics Workbench were run on a Dell Precision with two Intel Xeon, 8 Hexacores and 128 GB RAM shared by Windows 7 64-bit and a Linux Mint 12 64-bit virtual machine (VirtualBox v. 4.2.6). To verify whether expression value differences after RNA amplification and sequencing were in line with original expression values, we performed quantitative PCR for ZIC2 (HS00600854_m1), ZIC5 (HS00741567_m1) and MYT1 (HS01027966_m1) in the stock solutions, which all showed comparable RNA read counts.

### Immunohistochemistry

Tissue slides (5 μm) were mounted on aminoacetylsilane coated glass slides (Starfrost; Berlin, Germany), deparaffinised in xylene and rehydrated in ethanol. Endogenous peroxidase activity was blocked by 1% hydrogen peroxide in methanol for 20 minutes. Microwave (700 W) pretreatment in tris(hydroxymethyl)-aminomethane-EDTA (pH 9.0) was performed for 15 minutes. Slides were incubated with primary antibody targeting HELLS (1:500, clone H4, sc46665, Santa Cruz, Germany), ZIC2 (1:1000, ab15392, Merck Millipore, Amsterdam, The Netherlands) and ZIC5 (1:100; ab115566, Abcam, Cambridge, UK) followed by chromogenic visualization using the EnVision DAKO kit (DAKO, Glostrup, Denmark). Antibody specificity was tested using Western blotting and immunohistochemistry of positive and negative control tissues indicated by the manufacturer›s product sheets as well as the Human Protein Atlas www.proteinatlas.org. After counterstaining with hematoxylin, slides were thoroughly washed, dehydrated, cleared in xylene and mounted in malinol (Chroma-Geselschaft, Körgen, Germany). Antibody expression was scored by one investigator (MH) in a blinded setting. Cellular location, percentage of positive tumor cells and staining intensity were determined for all antibodies. Staining intensity was scored as negative (0; no staining), weak (1+; only visible at high magnification), moderate (2+; visible at low magnification) and strong (3+; striking at low magnification).

### Statistics

For each immunohistochemical parameter, the average of the tissue microarray scores was calculated per patient. Statistical associations between marker expression and continuous clinicopathologic parameters (age and PSA at time of diagnosis) were analyzed using Student's *t*-test, and with categorical parameters (Gleason score, pT-stage and surgical margins) using Pearson's Chi-square (Χ2) test. To determine whether expression was predictive for biochemical recurrence, we used uni- and multivariate Cox regression with stepwise backward entering of covariates. A two-sided *p*-value < 0.05 was considered significant. All statistics were performed using SPSS 20 (SPSS; Chicago, USA).

## SUPPLEMENTARY MATERIALS TABLES


