# Supplementary material for: Gene-expression analysis of gleason grade 3 tumor glands embedded in low- and high-risk prostate cancer
Source: Oncotarget. 2016 May 13;7(25):37846–56. doi: 10.18632/oncotarget.9344 (PMC5122354; doi:10.18632/oncotarget.9344)
Supplement: Supplementary file 1 [file oncotarget-07-37846-s001.pdf]

Gene-expression analysis of gleason grade 3 tumor glands embedded in low- and high-risk prostate cancer

Supplementary Materials

Supplementary Table S1: List of genes over-expressed in Gleason grade 3 tumor glands being part of a Gleason score 4 + 3 = 7 as compared to Gleason score 3 + 3 = 6

Gene	logFC	logCPM	*p*−value
GLYATL3	−9,36	−1,72	0,00
CDC20B	−8,71	−2,86	0,00
ZIC1	−8,53	−3,48	0,00
COX7B2	−8,11	−3,01	0,00
HORMAD2	−8,07	−4,04	0,00
ENTHD1	−7,95	−4,03	0,00
FEZF2	−7,62	−4,74	0,00
IBSP	−7,39	−4,95	0,01
DYTN	−7,39	−4,68	0,00
MAGEC2	−7,36	−4,69	0,00
OR8A1	−7,16	−4,17	0,00
SLC17A4	−7,16	−3,74	0,00
NXPH1	−7,12	−5,02	0,00
PDIA2	−7,03	−4,78	0,00
PNLIPRP3	−6,98	−4,17	0,00
ZIC4	−6,88	−5,05	0,01
CYP1A2	−6,88	−4,23	0,00
OR8D1	−6,78	−4,47	0,00
HOXC13	−6,47	−4,68	0,00
CREG2	−6,46	−0,56	0,00
CHODL-AS1	−6,40	−4,77	0,01
OR52N2	−6,40	−5,10	0,00
DMP1	−6,33	−5,91	0,04
LILRA4	−6,25	−5,74	0,01
MUC6	−6,25	1,69	0,00
PTX4	−6,23	−2,38	0,00
AFP	−6,17	−5,24	0,01
CCDC144NL	−6,17	−5,67	0,01
TAG	−6,17	−5,16	0,01
CEACAM7	−5,99	−5,49	0,01
SPATA21	−5,99	−5,47	0,00
KCNH7	−5,98	−0,12	0,00
ANKUB1	−5,89	−6,08	0,04
PADI3	−5,89	−5,43	0,02
MUC21	−5,89	−5,60	0,01
ANKRD30A	−5,89	−1,77	0,00

KLRC2	−5,79	−6,23	0,04
H1FNT	−5,79	−6,07	0,03
PLA2G4B	−5,79	−5,85	0,02
MAGEC1	−5,79	−5,71	0,02
PPP1R42	−5,79	−5,77	0,01
DIRAS2	−5,77	−3,18	0,00
FGF20	−5,68	−6,27	0,05
OR8G5	−5,68	−5,55	0,02
TPTE	−5,68	−5,55	0,02
GCM1	−5,68	−5,85	0,02
RGS6	−5,68	−5,31	0,01
KCNV1	−5,55	−5,68	0,02
SSX1	−5,55	−5,68	0,02
INSL3	−5,55	−6,19	0,02
RPRML	−5,55	−5,66	0,01
CNPY1	−5,42	−5,83	0,03
IL36RN	−5,42	−6,01	0,03
BARX1	−5,42	−5,65	0,02
LHX2	−5,42	−5,96	0,01
MAGEA8	−5,32	−3,54	0,00
HNF1A-AS1	−5,27	−6,09	0,05
DCAF4L2	−5,27	−5,99	0,04
DHH	−5,27	−6,06	0,03
INSL6	−5,27	−5,69	0,03
ABRA	−5,27	−5,94	0,01
TNNI3	−5,10	−6,18	0,05
CHRM4	−5,10	−6,64	0,04
TRPC3	−5,10	−6,55	0,04
KRT73	−5,10	−6,16	0,04
TDO2	−5,10	−1,44	0,00
NETO1	−5,02	−2,70	0,00
PPP3R2	−5,02	−2,73	0,00
CFHR4	−4,92	−6,54	0,05
IL9R	−4,92	−6,33	0,04
FAM92B	−4,92	−6,68	0,03
IFNG	−4,92	−6,64	0,02
TUBA4B	−4,80	−4,78	0,01
WDR72	−4,78	−2,60	0,00
TRDN	−4,78	−2,27	0,00
PRRG3	−4,70	−6,16	0,03
GRIK4	−4,66	−4,41	0,01
LY6D	−4,58	−1,75	0,00
SLC4A8	−4,49	−3,19	0,00
HSPA6	−4,46	−1,21	0,00
CNTNAP5	−4,45	−5,14	0,01
CYP1A1	−4,45	−4,42	0,00

LRRIQ4	−4,39	−5,32	0,03
PROK2	−4,39	−4,21	0,00
ACCN1	−4,38	−4,59	0,00
MYT1	−4,31	−4,33	0,00
METTL7B	−4,28	−3,31	0,00
ZNF804B	−4,26	−3,53	0,00
OLFM4	−4,25	4,36	0,00
OMD	−4,23	1,41	0,00
TDRD9	−4,19	−3,40	0,01
KLK14	−4,13	−2,40	0,00
SLC18A3	−4,13	−5,55	0,04
TULP2	−4,08	−2,96	0,00
ZIC5	−4,08	−1,00	0,00
ATP12A	−4,05	−5,46	0,02
LINC00307	−3,98	−5,02	0,01
GABRA2	−3,98	−5,33	0,00
TTC29	−3,96	−4,82	0,00
HAVCR1	−3,90	−5,02	0,01
SLC13A1	−3,81	−4,95	0,01
ACSM5	−3,81	−5,28	0,00
POU5F1P4	−3,80	−2,14	0,01
XIRP1	−3,77	−4,69	0,00
HAO1	−3,70	−1,96	0,00
PHOSPHO1	−3,62	−5,40	0,03
HOXB7	−3,54	−4,46	0,01
SLC6A17	−3,50	−1,07	0,00
PON1	−3,49	0,43	0,00
KRTAP13-2	−3,48	−0,42	0,01
SLC6A15	−3,42	−1,73	0,00
RHCG	−3,38	−4,20	0,01
CA1	−3,38	−4,08	0,02
EFNA2	−3,36	−3,90	0,00
DLEU7	−3,35	−4,29	0,03
KRT2	−3,34	−4,42	0,00
IGSF21	−3,32	−0,02	0,01
SLC6A20	−3,28	−5,27	0,02
FAM176A	−3,28	−4,80	0,00
CXCL9	−3,25	1,72	0,00
DSCAM-AS1	−3,22	−0,86	0,00
HIST1H2AB	−3,14	−6,05	0,04
CDHR4	−3,13	−5,15	0,05
HOXB9	−3,12	−2,48	0,01
PLAT	−3,12	2,65	0,00
FGF12	−3,11	0,59	0,00
HCN4	−3,07	−4,97	0,01
MKRN3	−3,06	−3,18	0,01
KRT13	−3,06	0,95	0,01

NBEAP1	−3,06	0,82	0,00
KIAA1239	−3,06	−4,56	0,05
DNAH3	−3,05	−3,15	0,01
ZIC2	−3,05	−0,64	0,00
SCML4	−3,02	−4,32	0,01
UNC5A	−3,00	0,91	0,00
DDC	−2,98	−2,65	0,00
TMEM63C	−2,97	−2,26	0,03
HOGA1	−2,97	−5,28	0,01
FOXN4	−2,96	−3,50	0,04
PCDHB3	−2,96	1,18	0,00
ZNF536	−2,94	−3,29	0,00
GRIN3A	−2,92	2,08	0,00
CXCR2	−2,88	−5,35	0,02
SLC22A14	−2,88	−5,21	0,02
FOXS1	−2,85	−4,44	0,01
TMPRSS4	−2,84	−1,80	0,01
TNNT1	−2,83	−4,43	0,01
CXCL6	−2,82	−3,61	0,00
SLPI	−2,82	−0,54	0,00
ST6GALNAC5	−2,80	−0,56	0,00
SLC22A10	−2,79	−2,13	0,00
IL12RB1	−2,78	−4,38	0,04
BMX	−2,78	−4,59	0,01
B3GALT5	−2,77	−3,97	0,03
GPR158	−2,76	0,70	0,00
NMNAT2	−2,76	−0,30	0,00
DCLK1	−2,76	0,76	0,00
HOXC4	−2,72	−0,44	0,00
SI	−2,68	2,37	0,00
CA4	−2,68	−3,83	0,01
WNT10B	−2,67	−5,59	0,01
NLGN4X	−2,65	0,14	0,04
KRT4	−2,63	−1,89	0,02
VWA7	−2,61	−3,10	0,00
PAX1	−2,60	−3,47	0,04
CES3	−2,59	−1,37	0,00
UTS2D	−2,58	−2,13	0,02
DHRS2	−2,57	−3,62	0,00
TEKT1	−2,57	−4,93	0,03
HAL	−2,56	−5,82	0,03
ZBBX	−2,54	−3,52	0,03
UGT8	−2,53	−3,78	0,00
UPB1	−2,52	−4,56	0,01
EEF1A2	−2,52	0,02	0,00
CGA	−2,52	−2,92	0,04
MMP13	−2,51	−3,46	0,03

SIGLEC1	−2,51	−2,60	0,00
CAPN14	−2,50	−3,55	0,00
SPON2	−2,47	5,98	0,00
DUOX2	−2,47	−0,66	0,01
SCG5	−2,46	−2,50	0,04
PTGFR	−2,45	−1,85	0,00
CORT	−2,44	−4,97	0,02
COL12A1	−2,43	7,01	0,00
CCDC141	−2,39	1,38	0,00
CCL4	−2,38	−2,42	0,00
TMPRSS11A	−2,36	−3,94	0,04
TSPO2	−2,35	−5,47	0,05
NPPC	−2,35	−2,81	0,00
TLR8	−2,32	−4,18	0,02
MUC4	−2,31	1,14	0,00
SHCBP1	−2,31	−3,33	0,00
CCL3	−2,30	−2,22	0,01
AK5	−2,28	1,24	0,00
SLC16A6	−2,26	−2,00	0,01
HOXD-AS1	−2,25	−0,85	0,01
GRM7	−2,25	−3,17	0,02
TLE6	−2,25	−5,27	0,04
RORB	−2,24	0,33	0,00
ALB	−2,23	−1,93	0,00
CDT1	−2,23	−2,14	0,03
NTNG1	−2,23	0,84	0,00
E2F2	−2,23	−2,55	0,00
E2F7	−2,22	−1,81	0,00
COL11A1	−2,22	−4,89	0,04
FOXJ1	−2,21	−2,03	0,01
CLCA4	−2,21	−2,30	0,02
SERPINA4	−2,20	−4,39	0,01
RS1	−2,19	−4,80	0,05
XIRP2	−2,18	−3,96	0,03
FBXL16	−2,17	−1,76	0,00
ZMYND10	−2,17	−2,58	0,01
SEMA3D	−2,16	1,87	0,00
KCNJ10	−2,16	−0,29	0,00
IL11	−2,15	−5,83	0,04
MUC13	−2,15	1,07	0,00
TPPP3	−2,14	−1,97	0,02
GCNT3	−2,14	−2,99	0,02
GMNC	−2,13	1,36	0,02
UCA1	−2,13	−4,78	0,05
SLC38A4	−2,12	0,59	0,00
GDF11	−2,10	1,45	0,00
LANCL3	−2,09	−2,59	0,01

COCH	−2,08	−1,05	0,01
ATP8A2	−2,08	1,42	0,01
SSTR1	−2,07	−0,05	0,02
PKHD1	−2,07	−0,12	0,01
LGALS2	−2,07	−4,75	0,02
NRP1	−2,06	2,74	0,00
TMED6	−2,03	−1,31	0,00
CCDC78	−2,02	−3,40	0,00
GRPR	−2,01	0,82	0,00
CDH10	−2,01	−0,56	0,00
BANK1	−2,01	3,61	0,00
LRRTM4	−2,00	−3,09	0,02
HNF1A	−2,00	−2,40	0,00
KRT20	−2,00	−2,87	0,01
MOGAT2	−2,00	−3,56	0,02
DLGAP5	−1,99	−1,13	0,00
ODZ1	−1,99	5,10	0,00
PPFIA2	−1,98	1,05	0,02
RDH5	−1,98	−4,58	0,02
KYNU	−1,97	−3,85	0,01
MCOLN3	−1,97	−1,23	0,02
LMX1B	−1,96	−1,74	0,00
NKG7	−1,96	−3,49	0,04
PYHIN1	−1,96	−3,37	0,01
OLR1	−1,95	−1,11	0,00
SDS	−1,95	−2,23	0,03
CCDC83	−1,95	−2,65	0,04
CSMD1	−1,95	−1,56	0,01
KLHL6	−1,93	−2,48	0,00
HOXC6	−1,93	0,93	0,00
SPATC1	−1,92	−4,40	0,00
PLK1	−1,92	−1,31	0,00
PIRT	−1,91	−3,47	0,03
COL11A2	−1,91	−3,68	0,02
MED12L	−1,90	1,50	0,00
PCDHA10	−1,89	−1,00	0,01
TREM2	−1,88	−2,58	0,01
RND1	−1,87	−1,16	0,00
RAB39A	−1,87	−2,06	0,00
E2F8	−1,87	−2,38	0,00
PMS2P5	−1,86	−5,24	0,05
MADCAM1	−1,86	−4,85	0,02
MAOB	−1,86	1,19	0,03
CLSPN	−1,85	−1,62	0,00
GJA3	−1,85	−2,34	0,02
CEP55	−1,84	−1,77	0,01
NUF2	−1,84	−2,91	0,03

HAGHL	−1,83	−3,10	0,05
CELSR3	−1,83	−0,40	0,00
FOXL2	−1,83	−2,77	0,02
HS6ST2	−1,81	−1,06	0,03
APOE	−1,81	0,89	0,00
RFX6	−1,80	−2,28	0,05
NAT8L	−1,80	−2,22	0,01
CADPS	−1,79	−0,21	0,01
MOB4	−1,79	−4,17	0,02
MT1DP	−1,79	−4,09	0,01
PTPRZ1	−1,79	−0,68	0,00
IL1RAPL1	−1,79	−0,54	0,01
PLTP	−1,78	2,00	0,01
PSMC3IP	−1,77	−2,48	0,00
DUSP8	−1,77	0,23	0,00
GAPT	−1,77	−3,85	0,02
OSM	−1,76	−5,23	0,03
GSG2	−1,74	−2,30	0,01
PBK	−1,74	−2,93	0,04
CCDC19	−1,73	−3,02	0,04
ULBP1	−1,73	−4,24	0,04
LINC00173	−1,73	−4,05	0,03
MYBL2	−1,73	−1,57	0,00
ONECUT2	−1,73	1,72	0,00
ASF1B	−1,72	−1,75	0,01
HS3ST1	−1,72	0,76	0,01
TMEM59L	−1,72	−2,77	0,03
RASAL3	−1,72	−2,79	0,00
ANKRD29	−1,71	−0,61	0,02
ESPL1	−1,71	−1,58	0,02
NEK2	−1,70	−2,25	0,02
FAM171B	−1,69	0,68	0,01
MND1	−1,69	−3,42	0,00
OSTalpha	−1,69	−0,84	0,03
GPRC5A	−1,69	3,39	0,03
GFI1	−1,68	−1,93	0,02
IL1RN	−1,67	−1,17	0,03
LINGO3	−1,67	−4,49	0,04
TNFSF8	−1,66	−2,19	0,00
JAG1	−1,66	3,04	0,00
APOC1	−1,66	0,62	0,01
CD22	−1,66	−2,63	0,01
GALNT13	−1,66	−1,80	0,04
SUSD2	−1,65	−0,69	0,00
CXCL11	−1,65	0,05	0,02
KIF18B	−1,65	−2,16	0,02
POLQ	−1,64	−2,18	0,01

PLA2G16	−1,63	0,63	0,01
GPR171	−1,62	−2,26	0,01
GABRP	−1,62	0,06	0,04
FOLH1	−1,61	4,67	0,00
MSR1	−1,61	0,38	0,00
BIRC5	−1,61	−1,20	0,01
MKI67	−1,61	2,64	0,00
ADAMTS3	−1,60	1,62	0,00
PTTG1	−1,60	−1,30	0,01
CD163	−1,60	−0,91	0,00
PHKG1	−1,58	−4,16	0,03
MUC1	−1,58	−0,08	0,01
NPY	−1,58	7,45	0,02
HAVCR2	−1,57	−1,90	0,01
AURKB	−1,57	−3,00	0,01
KCNJ12	−1,56	−2,67	0,03
LPPR4	−1,56	−2,17	0,01
CYP4Z2P	−1,55	−4,33	0,03
HMMR	−1,54	−0,05	0,00
VSIG4	−1,54	−1,78	0,00
TPX2	−1,53	0,57	0,00
EGR1	−1,53	6,27	0,00
DIAPH3	−1,53	−0,98	0,04
UFSP1	−1,53	−3,26	0,01
AGT	−1,52	−2,08	0,04
ITM2A	−1,52	−0,01	0,03
GTSE1	−1,52	−2,01	0,05
MCTP1	−1,51	−0,47	0,01
HEY2	−1,51	−0,40	0,01
PRR7	−1,51	−2,81	0,04
CN5H6.4	−1,50	−5,13	0,04
WNT5A	−1,49	2,32	0,02
DPH3P1	−1,48	−4,35	0,05
DPF1	−1,48	−4,29	0,03
EXO1	−1,47	−2,38	0,01
PTPRT	−1,47	2,06	0,00
STON2	−1,47	1,74	0,00
SIGLEC9	−1,46	−3,47	0,03
REEP1	−1,45	−0,80	0,01
PEAR1	−1,45	−1,81	0,01
ITGBL1	−1,45	−0,98	0,01
CDC45	−1,45	−2,89	0,01
MELK	−1,44	−0,97	0,01
NCAPG	−1,44	−1,04	0,03
CDK5R1	−1,44	−0,65	0,04
FKBP10	−1,43	1,18	0,03
P2RX5-TAX1BP3	−1,43	−3,43	0,04

RSPH4A	−1,43	−1,30	0,01
KIF20A	−1,43	−0,51	0,02
TOP2A	−1,42	1,90	0,00
PPP2R2C	−1,42	0,59	0,00
ZNF296	−1,42	−3,07	0,04
PTK6	−1,41	−2,78	0,02
DPY19L2	−1,41	0,32	0,02
WDR66	−1,41	−1,65	0,01
CERS1	−1,41	−2,45	0,02
A1BG-AS1	−1,41	−4,50	0,03
SPAG5	−1,40	−0,79	0,00
FLJ35946	−1,39	0,74	0,03
DTL	−1,39	−0,91	0,01
RYR2	−1,39	2,38	0,01
UMODL1	−1,39	−4,19	0,03
BUB1	−1,38	−0,60	0,01
MS4A7	−1,37	−0,71	0,00
LMNB1	−1,37	0,00	0,00
CDCA3	−1,37	−1,94	0,01
UBE2C	−1,36	−1,40	0,03
FLJ23867	−1,36	−0,38	0,03
DNASE2B	−1,34	1,47	0,04
PLEK	−1,33	−1,47	0,05
MAP2	−1,33	4,60	0,01
BICC1	−1,33	1,67	0,01
SLC22A31	−1,33	−3,85	0,04
CDKN2D	−1,32	−1,38	0,02
F5	−1,32	3,82	0,03
SLC6A1	−1,32	−4,36	0,04
SEMA3G	−1,32	−1,86	0,03
KIF23	−1,32	−0,71	0,01
MAP6D1	−1,32	0,08	0,04
PDE3A	−1,31	1,97	0,00
AATK	−1,31	−2,67	0,01
CDK1	−1,31	−1,69	0,02
GNB1L	−1,30	−3,25	0,02
AURKA	−1,30	−1,64	0,05
ZNF467	−1,30	−1,07	0,00
CCDC108	−1,29	−2,16	0,01
CIT	−1,29	−0,35	0,01
SNCA	−1,29	0,78	0,00
ZP1	−1,29	−1,08	0,02
MXD3	−1,29	−2,95	0,05
PITPNM1	−1,28	−0,48	0,00
LAG3	−1,28	−1,25	0,01
CENPF	−1,28	1,58	0,01
DISP2	−1,28	−1,58	0,02

IER5	−1,28	−1,94	0,03
VAX2	−1,28	−3,69	0,03
GPR179	−1,28	−3,11	0,02
ANO2	−1,26	−2,47	0,04
SLA	−1,26	−0,98	0,00
FAM111B	−1,26	−0,19	0,03
FAM55C	−1,26	−0,04	0,00
MLF1IP	−1,26	−0,04	0,00
AKAP12	−1,25	4,00	0,00
TERC	−1,25	0,41	0,01
TMEFF2	−1,24	6,82	0,00
CXCL12	−1,24	2,07	0,00
EPPK1	−1,24	1,01	0,02
MTUS2	−1,24	0,16	0,00
SEC16B	−1,23	−1,71	0,01
RTN1	−1,22	2,69	0,02
SLC10A5	−1,22	0,94	0,03
SPG20	−1,22	2,66	0,00
ASPM	−1,22	0,21	0,01
DRAM1	−1,21	0,31	0,01
HSPA1B	−1,20	2,95	0,01
KIF2C	−1,20	−2,47	0,04
RGS2	−1,20	3,13	0,02
FAM110B	−1,20	1,34	0,00
CASC5	−1,19	−0,19	0,02
ODC1	−1,19	6,68	0,01
RGS11	−1,19	0,09	0,00
GAS2L3	−1,18	−1,37	0,03
CYTH3	−1,18	2,17	0,01
EME1	−1,18	−2,86	0,02
FRY	−1,18	1,21	0,00
RAB3IL1	−1,18	−1,82	0,01
PCSK1N	−1,18	−0,37	0,01
PACSIN1	−1,17	−1,56	0,00
SKA3	−1,16	−1,05	0,03
PLCE1	−1,15	0,42	0,01
MDK	−1,14	2,95	0,00
RELN	−1,14	2,35	0,01
GMNN	−1,12	−0,37	0,02
MAPK8IP2	−1,12	−0,36	0,04
TACC3	−1,12	−0,92	0,03
CSF2RA	−1,11	−3,07	0,04
SKA1	−1,11	−1,59	0,02
CD68	−1,11	0,99	0,02
RASSF2	−1,10	0,57	0,02
CDR2	−1,10	2,77	0,04
GGH	−1,10	−1,00	0,04

BAMBI	−1,10	0,75	0,00
BARX2	−1,10	0,04	0,01
PLD1	−1,10	−0,37	0,01
IRF5	−1,10	−1,87	0,01
ADM	−1,09	0,29	0,04
SP6	−1,09	−2,97	0,03
TMEM204	−1,09	−0,59	0,02
TK1	−1,09	−0,09	0,01
ARSG	−1,08	−0,32	0,03
IFI30	−1,08	1,92	0,02
SLCO2B1	−1,08	0,01	0,00
SASH3	−1,08	−1,69	0,05
MMP11	−1,07	−2,46	0,01
E2F1	−1,07	−1,78	0,03
APOD	−1,06	5,60	0,05
WDR62	−1,06	−2,21	0,04
CPNE7	−1,05	−1,02	0,04
KIAA1683	−1,05	0,40	0,02
CMKLR1	−1,05	−0,88	0,04
ANKRD19P	−1,05	−2,40	0,04
PHLDA2	−1,04	−0,53	0,02
HELLS	−1,04	−0,47	0,01
GAL3ST4	−1,04	−0,84	0,02
FSD1L	−1,03	−0,07	0,01
CDON	−1,03	2,00	0,00
ARL9	−1,03	−2,83	0,04
BCMO1	−1,03	−2,55	0,04
CD86	−1,03	−2,60	0,02
FCGR2A	−1,02	0,34	0,00
CDKL3	−1,02	−2,41	0,05
CENPE	−1,02	−0,19	0,03
PRR11	−1,02	−0,67	0,02
MAPK4	−1,02	1,83	0,02
MAD2L1	−1,02	−1,79	0,02
RHBDL1	−1,02	−3,51	0,04
RANBP3L	−1,01	2,40	0,00
LPGAT1	−1,01	3,68	0,00
ERRFI1	−1,01	3,84	0,01
FBP2	−1,01	−1,66	0,05
SLC4A3	−1,01	−0,51	0,01
FGFRL1	−1,01	3,90	0,00
CDCA5	−1,01	−1,26	0,00
CRNDE	−1,00	−0,14	0,03

LogFC values represent 2log fold changes of RNA expression value of Gleason grade 3 in Gleason score 6 prostate cancer divided by its expression value in Gleason score 4 + 3 = 7 prostate cancer. Negative values represent up-regulation of genes in Gleason score 4 + 3 = 7 prostate cancer. LogCPM values represent 2log counts per million mapped reads.

Supplementary Table S2: List of genes over-expressed in Gleason grade 3 tumor glands being part of a Gleason score 3 + 3 = 6 as compared to Gleason score 4 + 3 = 7

Gene	logFC	logCPM	*p*−value
SST	8,51	−3,54	0,00
VIP	8,28	−3,76	0,00
SEMG1	7,92	−4,10	0,00
INSL5	7,59	−2,74	0,00
KIF25	7,53	−4,43	0,00
OR2T10	7,04	−4,93	0,01
ARSF	6,65	−5,21	0,01
SLC6A2	6,60	−5,20	0,01
FLJ46361	6,48	−4,99	0,00
PLA2G3	6,48	−5,46	0,01
MYH6	6,13	−4,98	0,01
GFI1B	6,05	−5,19	0,01
S100A5	5,96	−5,47	0,01
KLHL4	5,87	−5,90	0,01
SPZ1	5,87	−5,99	0,03
G6PC2	5,77	−5,49	0,00
KRT81	5,77	−5,79	0,01
GRM8	5,77	−5,79	0,01
WFIKKN2	5,77	−5,80	0,01
NHLRC4	5,77	−6,09	0,03
CARTPT	5,72	−3,29	0,00
FOXI1	5,67	−5,69	0,02
BCORP1	5,66	−3,87	0,00
OR1B1	5,56	−5,86	0,02
TWIST2	5,56	−6,10	0,02
HSD52	5,56	−5,96	0,03
CCDC164	5,56	−6,21	0,03
OR5P2	5,56	−6,22	0,04
PLA2G2F	5,43	−6,35	0,04
SLC25A31	5,43	−6,17	0,05
ORM1	5,43	3,05	0,00
NPY2R	5,39	−2,67	0,00
PGCP1	5,30	−6,02	0,02
CSDAP1	5,30	−6,06	0,03
BTNL2	5,30	−6,42	0,04
KCNB2	5,30	−6,47	0,04
OR2T8	5,15	−6,53	0,05
AGPAT4-IT1	5,15	−6,33	0,05
KCNK7	4,99	−6,63	0,05
COL9A1	4,93	0,42	0,00
HCG4	4,83	−4,17	0,00
TMCC2	4,80	−6,53	0,04
OR52E2	4,80	−6,40	0,05

OR7D2	4,75	−4,08	0,00
FRMPD4	4,69	−0,56	0,00
SLC35D3	4,62	−4,17	0,00
PAH	4,56	0,99	0,01
GPR116	4,51	2,59	0,00
STAC2	4,48	−4,24	0,01
MYL1	4,42	−3,64	0,03
FOLR1	4,39	−4,82	0,00
MPZ	4,39	−4,93	0,00
GABRG2	4,37	−4,32	0,02
NTF4	4,34	−5,14	0,02
VAT1L	4,34	−5,19	0,03
HCG23	4,23	−4,92	0,00
KCNK2	4,23	−5,13	0,01
DACH2	4,22	−1,31	0,00
PCDH17	4,21	3,28	0,00
GPR62	4,17	−5,26	0,02
GABRQ	4,17	−5,08	0,04
CPNE6	4,10	−4,09	0,00
UCN3	4,07	−3,91	0,01
IQSEC3	4,04	−5,12	0,01
KPNA7	3,98	−5,29	0,01
KCNH4	3,98	−5,48	0,04
CXorf57	3,93	−4,45	0,00
LINC00158	3,92	−1,47	0,00
SEMA5B	3,86	−1,62	0,00
NLRP14	3,84	−5,40	0,01
SLC2A5	3,80	−0,09	0,00
PTCHD3	3,76	−4,58	0,04
UPK1A	3,73	−1,10	0,01
HGF	3,73	0,47	0,00
FCRL3	3,71	−4,12	0,01
CYP27A1	3,70	1,85	0,00
HECW1	3,61	−0,01	0,00
DPYS	3,57	−1,13	0,00
ASCL2	3,54	−5,00	0,02
PAX8	3,53	−2,43	0,00
COL2A1	3,53	3,19	0,00
SLC7A10	3,50	−5,19	0,03
TAF7L	3,50	−5,69	0,04
KANK4	3,49	−3,30	0,00
KRT222	3,48	−4,74	0,03
PRDM8	3,48	0,68	0,00
KCNE3	3,46	−1,36	0,00
DEFB1	3,44	−4,21	0,00
KCNS1	3,38	−2,87	0,01

CD33	3,36	−4,40	0,00
CD177	3,36	3,31	0,00
CCK	3,35	−0,77	0,02
DRD1	3,33	−2,95	0,01
AP1B1P1	3,32	−2,83	0,00
PROK1	3,32	−1,57	0,00
OVCH2	3,28	−3,96	0,04
ORM2	3,27	1,12	0,01
TPTE2P1	3,26	−4,00	0,01
CIDEC	3,25	−4,78	0,01
FBXO17	3,25	−1,65	0,00
HOXD9	3,24	−3,40	0,00
TMEM132D	3,14	−1,61	0,01
CXCL5	3,13	−3,20	0,02
NRG2	3,13	−4,48	0,02
NAALADL1	3,10	−3,87	0,00
HOXD10	3,07	−3,52	0,02
OR2W5	3,06	−5,68	0,05
ZNF664-FAM101A	3,06	−5,09	0,02
TDRD12	2,98	−5,54	0,05
COL19A1	2,93	−6,08	0,05
CCDC8	2,92	−3,31	0,04
RNF112	2,92	−3,52	0,00
SLC38A3	2,91	−5,26	0,04
SOX2	2,90	−1,45	0,00
LRRN4	2,88	−2,71	0,00
SFTPA2	2,87	5,06	0,00
SLC22A1	2,85	−3,36	0,00
BCAT1	2,84	−0,19	0,00
GPX7	2,84	−1,05	0,00
NRG1	2,82	−1,73	0,00
CHODL	2,82	−3,66	0,00
ZNF503-AS1	2,82	−2,25	0,00
CYP3A43	2,78	−3,75	0,04
ZNF804A	2,78	−4,36	0,01
JPH2	2,78	−2,54	0,00
PMP2	2,76	−2,29	0,00
PCYT1B	2,76	−3,87	0,02
FAM106A	2,72	−4,57	0,02
EFHD1	2,72	−0,34	0,01
NEFH	2,71	5,48	0,01
THEG5	2,71	−1,70	0,00
SLC16A5	2,66	−0,87	0,00
FLRT3	2,66	1,18	0,00
MBOAT4	2,65	−5,51	0,05
OR2C3	2,64	0,60	0,00

PIK3C2G	2,59	−3,20	0,02
OR1L8	2,59	−4,32	0,00
ATCAY	2,57	−4,37	0,04
COL17A1	2,56	0,18	0,00
SLC12A3	2,53	−3,94	0,01
APOB	2,53	−4,49	0,03
FABP4	2,53	−3,46	0,01
FAM83B	2,52	−1,07	0,00
VSNL1	2,52	−1,89	0,02
P2RX2	2,51	−3,68	0,00
GAP43	2,51	−4,59	0,01
PTPRN	2,51	−4,37	0,04
KCNQ5	2,51	−1,84	0,01
LINC00202	2,50	−3,24	0,02
DMKN	2,49	−2,23	0,00
SULT1C2P1	2,47	−1,20	0,01
CD28	2,46	−0,05	0,00
RND2	2,46	−3,32	0,00
NUDT9P1	2,45	−5,15	0,02
HOXD11	2,44	−1,75	0,04
BPIFB2	2,40	1,72	0,00
SGCA	2,39	−4,21	0,01
ACTC1	2,39	−2,66	0,02
HIF3A	2,38	−2,26	0,02
ZNF208	2,38	−2,89	0,00
TRIM9	2,37	−5,20	0,02
CTF1	2,35	−2,20	0,01
DKK3	2,34	1,53	0,00
SYTL5	2,33	−1,30	0,00
AJAP1	2,32	−4,21	0,01
BMPER	2,30	−3,16	0,01
CRIP3	2,29	−5,36	0,05
VSIG2	2,27	1,91	0,02
DIRAS3	2,25	−3,16	0,03
LURAP1	2,24	−4,17	0,03
LINC00327	2,23	−1,84	0,00
USP44	2,22	−3,19	0,01
KRT5	2,22	1,44	0,03
ACSS3	2,20	−0,34	0,00
MCF2	2,20	−5,00	0,04
SLC22A3	2,18	4,43	0,00
KCNMB1	2,18	−0,12	0,00
KRT17	2,18	−0,09	0,04
TLR4	2,17	2,47	0,00
CYS1	2,17	−3,08	0,05
IL3RA	2,17	−3,96	0,02

CPLX3	2,16	1,98	0,02
ADH4	2,16	−3,15	0,00
CYP2C8	2,16	−4,33	0,03
SLC5A8	2,16	−2,59	0,01
CCR6	2,16	−4,33	0,03
LRP2	2,15	−2,54	0,03
RBFOX3	2,15	−2,07	0,01
SRD5A2	2,14	−0,85	0,00
PCDH7	2,14	1,02	0,00
ANKRD20A8P	2,13	−3,36	0,02
DLK2	2,12	−1,79	0,02
ABCC9	2,10	1,21	0,01
TAS2R10	2,09	−4,79	0,02
POF1B	2,09	0,34	0,02
CDH6	2,09	−1,80	0,05
RNF157	2,09	2,61	0,00
ANO5	2,08	−1,17	0,01
ZNF215	2,08	−1,72	0,05
SNCAIP	2,08	−2,37	0,02
PAK3	2,07	−1,79	0,02
ZNF90	2,07	−2,70	0,03
OR2L13	2,07	−4,07	0,02
GPX3	2,07	3,03	0,00
ATP1A2	2,06	−1,27	0,00
ADRA1A	2,06	−2,85	0,02
ZNF781	2,05	−3,87	0,02
SLC14A1	2,05	2,37	0,02
RDH16	2,05	−0,04	0,04
TP63	2,05	1,02	0,05
SOX30	2,04	−3,48	0,02
KIAA1210	2,03	0,53	0,01
CYP26B1	2,02	−2,65	0,02
NTRK3	2,02	−1,57	0,00
ACOT11	2,00	−2,82	0,02
AXDND1	2,00	−3,04	0,00
PDPN	1,97	−1,50	0,00
PPP4R1L	1,96	−2,35	0,02
NKD2	1,95	−4,39	0,02
CCDC81	1,95	−3,07	0,01
CELF4	1,94	−2,66	0,01
ZNF257	1,94	−2,18	0,03
BMP7	1,93	0,09	0,03
FAM3D	1,92	0,02	0,00
CNGA4	1,90	−4,41	0,05
NDNF	1,89	0,55	0,01
SIX2	1,88	−3,62	0,03

ATP6V0D2	1,88	−3,32	0,03
SMOC1	1,88	−2,04	0,03
LAMP5	1,87	−0,47	0,04
MPP2	1,86	−3,30	0,04
ACTG2	1,85	4,76	0,00
MACROD2-AS1	1,84	−3,31	0,02
WISP2	1,84	−4,52	0,01
ZNF711	1,83	−0,81	0,01
BCL2L15	1,83	−1,85	0,04
HSPB8	1,82	−0,30	0,01
RNF165	1,82	−1,85	0,04
CHRDL1	1,82	1,27	0,00
PTN	1,81	4,46	0,00
ASB5	1,81	−0,09	0,01
DNM1	1,80	−2,78	0,05
ZNF454	1,80	−1,21	0,01
CDH3	1,80	1,34	0,00
SLITRK5	1,77	−1,26	0,01
VIT	1,77	−3,57	0,03
GTF2IRD2B	1,77	−4,10	0,01
SOCS1	1,76	−1,17	0,05
DCHS1	1,75	2,85	0,00
ZNF300P1	1,75	−1,49	0,03
CDCA7	1,73	−0,39	0,00
LAYN	1,72	−2,41	0,04
SRPX	1,71	−1,71	0,01
SULT4A1	1,71	−4,84	0,03
PGM5	1,71	1,02	0,00
WFDC1	1,70	−1,71	0,04
MYL9	1,70	3,16	0,00
LSAMP	1,69	2,80	0,04
ISL1	1,69	−1,73	0,01
ADAMTS5	1,69	−2,29	0,02
SLC5A4	1,68	0,73	0,00
TMEM35	1,68	−3,03	0,03
MET	1,68	0,50	0,02
HCAR1	1,67	1,20	0,00
MYOCD	1,65	−0,02	0,01
LGI4	1,65	−3,23	0,02
RIN3	1,65	−0,40	0,02
GRIK5	1,64	−2,86	0,02
PKD2L2	1,64	−3,61	0,01
TBX5	1,64	−2,71	0,05
PDZRN4	1,63	−1,60	0,01
RFPL2	1,63	0,71	0,00
RAPGEF4	1,62	2,65	0,00

PCP4	1,61	0,82	0,01
HCAR3	1,60	−4,32	0,03
SERPINF1	1,60	0,73	0,00
PHYHIPL	1,60	−1,34	0,02
P2RX1	1,59	−3,26	0,05
SPEG	1,58	−2,18	0,03
NRK	1,58	−1,74	0,01
IL5RA	1,58	0,77	0,00
FAM86B1	1,57	−2,56	0,02
PTGIS	1,57	−0,10	0,00
IGFBP6	1,56	−0,74	0,00
SERTAD4	1,56	−1,53	0,03
SLC16A7	1,56	−2,84	0,02
ST6GALNAC4	1,56	−3,54	0,04
IL33	1,56	−0,89	0,03
KCNU1	1,55	−3,20	0,02
FERMT1	1,55	2,19	0,00
MAGI2-AS3	1,54	−1,34	0,04
TGFB1I1	1,53	−0,37	0,03
GCG	1,53	−1,42	0,03
PLCL2	1,53	−2,85	0,05
PLIN2	1,53	1,96	0,00
KLRG1	1,53	−2,37	0,04
PZP	1,53	−2,66	0,02
CYP3A5	1,53	1,06	0,03
WIF1	1,52	−3,16	0,02
ABCA6	1,52	−1,06	0,00
ACTA2	1,52	4,38	0,00
ID4	1,52	−0,40	0,01
GALNT14	1,51	−3,17	0,04
FAM133B	1,51	−3,66	0,03
ADH6	1,50	−1,83	0,00
FUT9	1,48	−1,57	0,02
NINL	1,48	0,33	0,01
CNN1	1,48	1,75	0,01
PHACTR1	1,48	−2,21	0,01
MAGI2	1,48	1,31	0,01
RAB9B	1,48	−1,85	0,01
FGF7	1,48	−1,02	0,03
EFEMP1	1,47	0,66	0,00
FLNA	1,47	3,94	0,00
FAT3	1,47	0,32	0,01
SYT6	1,46	−0,04	0,00
FMO4	1,46	−0,65	0,03
MYOF	1,45	3,40	0,01
FLG2	1,45	−3,48	0,02

UCHL1	1,45	−1,17	0,04
BOC	1,45	1,33	0,01
ZNF843	1,44	−3,92	0,03
MAML2	1,44	2,98	0,01
ACSM3	1,44	3,04	0,00
PTGDS	1,43	1,54	0,00
KLHL10	1,43	−4,72	0,05
FREM2	1,42	−0,21	0,01
SP140L	1,42	−0,74	0,04
FAIM2	1,42	−1,55	0,01
COL25A1	1,41	0,62	0,00
CCL2	1,41	1,91	0,03
TPM2	1,40	3,31	0,00
TSC22D1-AS1	1,40	−3,07	0,03
LDB3	1,39	−0,79	0,03
NOV	1,39	2,47	0,00
MYH11	1,39	5,90	0,00
IGFBP5	1,38	4,34	0,03
CYP1B1	1,38	5,20	0,00
ADPRHL1	1,37	0,55	0,03
FLNC	1,37	1,24	0,00
SEMA7A	1,36	−3,38	0,04
NT5E	1,36	−1,23	0,02
PRH2	1,36	−1,76	0,01
PRKY	1,34	0,36	0,00
ZNF185	1,34	1,28	0,00
MYOM1	1,34	−1,61	0,01
POU5F2	1,33	−3,20	0,03
ZNF774	1,33	−1,03	0,01
SCNN1A	1,33	3,05	0,01
FLJ10661	1,32	−3,61	0,05
NCKAP5	1,31	2,26	0,00
NUDT18	1,30	−2,31	0,03
MSRB3	1,29	0,97	0,01
TAGLN	1,29	3,19	0,00
LEPREL2	1,29	−1,66	0,01
PTRF	1,29	2,53	0,04
CNN3	1,29	4,08	0,00
NRCAM	1,29	2,16	0,02
ZNF469	1,29	−1,79	0,02
ESR2	1,28	−3,21	0,01
RPA4	1,28	−3,66	0,05
MRVI1	1,28	0,83	0,02
MARVELD1	1,27	0,15	0,01
NHS	1,27	−0,06	0,01
HOTTIP	1,27	1,28	0,02

ID2	1,27	2,12	0,03
CSPG4	1,27	−0,83	0,02
CDH26	1,26	2,58	0,00
PATE4	1,26	−1,73	0,01
LMOD1	1,26	1,89	0,02
MCAM	1,26	−1,19	0,04
ZNF578	1,26	−0,14	0,01
CCDC3	1,25	−1,14	0,04
ZNF655	1,24	3,07	0,00
RND3	1,24	0,50	0,04
BNIPL	1,24	−2,16	0,03
TGFB3	1,23	2,65	0,01
EML5	1,23	2,38	0,00
RGNEF	1,22	0,34	0,04
RGS7BP	1,22	−0,93	0,03
SLC22A17	1,22	0,91	0,03
AOC3	1,22	0,87	0,02
MYLK	1,22	4,02	0,01
CORO2B	1,21	−2,88	0,02
MSTN	1,21	−2,98	0,05
TTTY10	1,21	−1,78	0,02
SCRG1	1,20	−3,26	0,04
DFNB59	1,19	−3,32	0,04
SYNPO2	1,19	4,28	0,01
LPXN	1,19	1,21	0,02
ANKRD31	1,19	−2,58	0,04
DES	1,18	2,94	0,01
PASK	1,18	1,27	0,02
SFN	1,18	1,80	0,01
COLQ	1,18	−2,15	0,02
FBLN1	1,17	2,67	0,02
CNTN1	1,17	0,00	0,01
PDLIM4	1,16	0,58	0,02
COTL1	1,15	2,65	0,02
PCDHB19P	1,15	−1,89	0,01
APLF	1,14	−0,83	0,04
SYNM	1,14	3,27	0,02
UCP2	1,14	1,56	0,04
SERPING1	1,12	3,19	0,00
AHNAK2	1,12	0,27	0,02
BCAS1	1,11	4,00	0,02
ARSJ	1,10	1,25	0,00
CPAMD8	1,09	1,11	0,03
GNG4	1,07	3,65	0,02
DLL1	1,06	0,12	0,04
CACNB4	1,05	0,61	0,04

GSTA4	1,05	2,69	0,00
HERPUD1	1,04	5,70	0,00
LEF1	1,04	1,24	0,01
FAT4	1,04	0,43	0,04
NPAS2	1,03	3,97	0,00
CHL1	1,03	2,44	0,05
SPARCL1	1,03	3,94	0,02
TMEM200A	1,03	−0,74	0,01
TNS1	1,02	4,43	0,03
MXRA7	1,01	1,97	0,02

LogFC values represent 2log fold changes of RNA expression value of Gleason grade 3 in Gleason score 6 prostate cancer divided by its expression value in Gleason score 4 + 3 = 7 prostate cancer. Positive values represent up-regulation of genes in Gleason score 3 + 3 = 6 prostate cancer. LogCPM values represent 2log counts per million mapped reads.
